# Influence of a special training process on the psychomotor skills of cadet pilots – Pilot study

**DOI:** 10.3389/fpsyg.2022.1019495

**Published:** 2022-09-29

**Authors:** Adam Prokopczyk, Zbigniew Wochyński

**Affiliations:** ^1^Department of Sport and Defence Education, Ponan University of Physical Education, Poznań, Poland; ^2^Department of Aviation Safety Transport, Military University of Aviation, Dęblin, Poland

**Keywords:** psychomotor skills, motor skills, sensoric, special training, diagnostic and training device, special aviation gymnastics instruments

## Abstract

**Objectives:**

The aim of the pilot study was to check the influence of the training process on the Special Aviation Gymnastics Instruments (SAGI) on the improvement of the psychomotor skills, expressed as an increase in the percentage of ability to perform all tasks and the number of reels on a loop.

**Materials and methods:**

Cadets - second year pilots (*n* = 20), male, mean age 20.8 years old, studying at the faculty of a pilot. Cadets were carrying out a 40-h special pilot training program on SAGI. They were subjected to two exercise tests (reels forward on looping), before and after the period of special training. Exercise tests were performed with the use of a diagnostic and training device used to assess psychomotor skills. During two tests, heart rate (HR) and blood pressure were measured. The obtained results were analysed statistically.

**Results:**

There was a statistically significant increase in the percentage of ability to perform all tasks (*p* < 0.01) and a statistically insignificant increase in the number of reels forward on looping, in test II in relation to test I. A significant increase was found in the correct execution of arithmetic operations (*p* < 0.05) in test II in relation to test I. In the remaining tests, an increase in results in test II was noted, but it was not statistically significant. There was a significant correlation between the percentage ability to perform all tasks and the number of completed reels in test I (*p* < 0.05) and insignificant in test II. In test II, a statistically insignificant higher level of heart rate and blood pressure before and after the effort was noted, compared to test I.

**Conclusion:**

It was found that the training process on SAGI increased the psychomotority level by increasing the percentage of ability to perform all tasks and the number of reels, in test II in relation to test I.

## Introduction

The process of special pilot skills preparation is a very important factor in modern flight preparation (Alexander and Stead, 2018). It is aimed at preparing the pilot for functioning and effective performance of complex and demanding tasks in the pilot working environment (Carretta, 2000; Li et al., 2005). As shown in other studies, for the conduct of an air mission by a pilot, required is appropriate level of preparation giving that allows possibility of processing a large amount of sensory information (Paśko et al., 2022). The level of this preparation is one of the determinants of the level of safety and effectiveness of the flight mission. Working environment of a military pilot requires a high level of psychomotor skills (McMahon and Newman, 2015). Researchers are studying the issues of psychomotorism for several dozen years. Despite many changes, testing of this feature is still present in modern training and selection systems for military pilots (Clem, 2020). Psychomotor skills is a motor activities that involve a significant perceptual and response load (Chaiken et al., 2000). The work of a military pilot requires highly specialized preparation including response time (Temme et al., 1995; Griffin and Koonce, 2009), information processing efficiency and motor skills - airplane operator activities (Astani and Macarie, 2013). Moreover, his psychophysical predispositions are very important, giving him high tolerance to negative flight factors (mainly acceleration; Wojtkowiak, 1989; Street Jr and Dolgin, 1994; McMahon and Newman, 2018), neurosensory predispositions, his level of efficiency and physical skills (Wochyński et al., 2010a; Kattenbach, 2017; McMahon, 2019). Due to such a wide range of skills required from a pilot, it is necessary to monitor the effects of the training process and modify it if necessary. Until now, Düffoure apparatus has been used most frequently to check the level of the psychomotor skills which was a result of the effectiveness of the military pilot training process. It was used to determine the level of visual-motor coordination before and after training on the Special Aviation Gymnastics Instruments (SAGI), including: gyroscope, Rhine wheel and looping (Kobos et al., 1994). The appearance and structure of these instruments is presented in other scientific papers (Wochyński and Sobiech, 2014, 2015, 2017). One of the most important elements of pilot training in preparation for flight is to achieve a habituation in psychomotor skills, high acceleration tolerance and spatial orientation (Wochyński et al., 2010b).

Taking into account the specificity of pilot’s work and the tasks facing him, it is important from the point of view of work efficiency to assess the psychomotor response during the exercises, and not only before and after the training. Therefore, in the present study, a diagnostic and training device was used to assess the level of psychomotority during physical effort. This test involves the use of complex visual-motor stimuli and the need to answer questions located in the central field of vision (in a specific time standard), while performing specific exercises (reels forward on the looping). The application of this test gives an opportunity to assess the ability to respond appropriately to a rapidly changing situation in the working environment of a military pilot (Glicksohn and Naor-Ziv, 2016). Psychomotor efficiency at the level of visual - motor coordination can be manifested in the reaction time parameters, i.e., the speed and correctness of their implementation (making a mistake; McMahon, 2019). Response errors may arise from a disturbance between stimulus and response, in a specific time standard. The test of visual - motor coordination efficiency during physical exertion is similar to the working conditions of a pilot during an air mission. Therefore, the authors undertook research on cognitive processes within in a specific time standard using the psychomotor test in cadets pilots during physical effort.

### Objectives

The aim of the pilot study was to check the influence of the training process on the Special Aviation Gymnastics Instruments (SAGI) on the improvement of the psychomotor skills. In this pilot study the authors put forward a hypothesis that the training process on SAGI will improve the level of psychomotor skills, measured with a diagnostic and training device, through a percentage increase in the ability to perform tasks (answers to questions in the central field of vision) and the level of motor skills (the number of reels made forward on the loop), in the sample after the training process is completed in relation to the sample before the process begins. Moreover, the authors asked a research question whether the improvement of psychomotor skills after the training process will be confirmed by lowering the correlation between the percentage ability to complete the task [%] and number of completed reels on the looping?

## Materials and methods

### Participants

The test included 20 cadets - pilots, second year, male, studying at the faculty of a military pilot, at the Military University of Aviation in Dęblin. The average age of the respondents is 20.8 years. The cadets implemented a special educational program, based on the Special Aviation Gymnastics Instruments (SAGI), to improve the level of psychomotority (Wochyński et al., 2010a). The test was carried out without a control group, due to the difficulty and specificity of the exercises included in the implemented test.

### Heart rate and blood pressure

Cadets had the heart rate and blood pressure measured before and immediately after the test, at the beginning and at the end of the training process. The measurement was performed with the heart rate and blood pressure measuring device, Microlife AG, type BP A2 Basic.

### Description of the test

**Figure 1:**
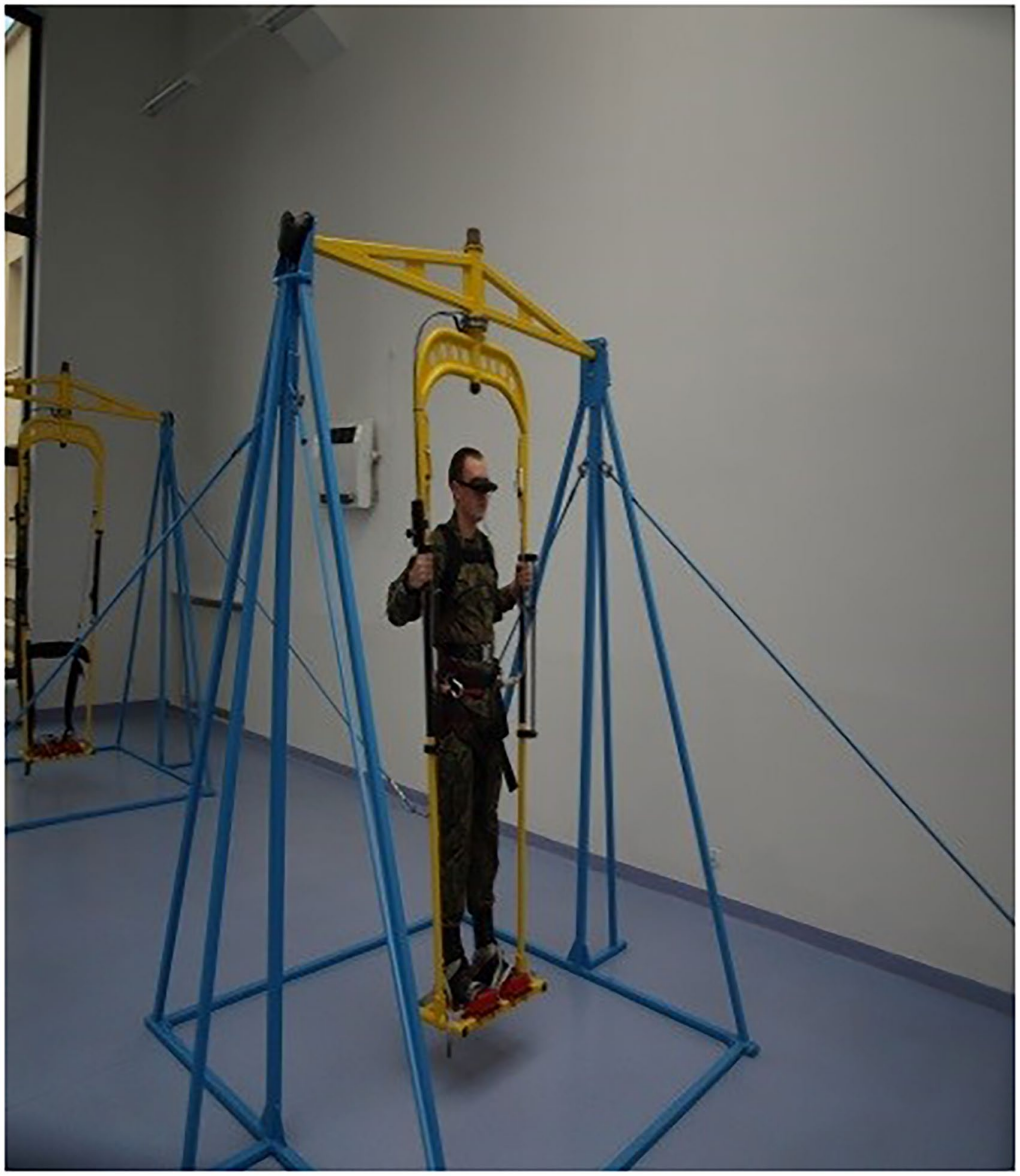
The test person with diagnostic and training device.

**Figure 2:**
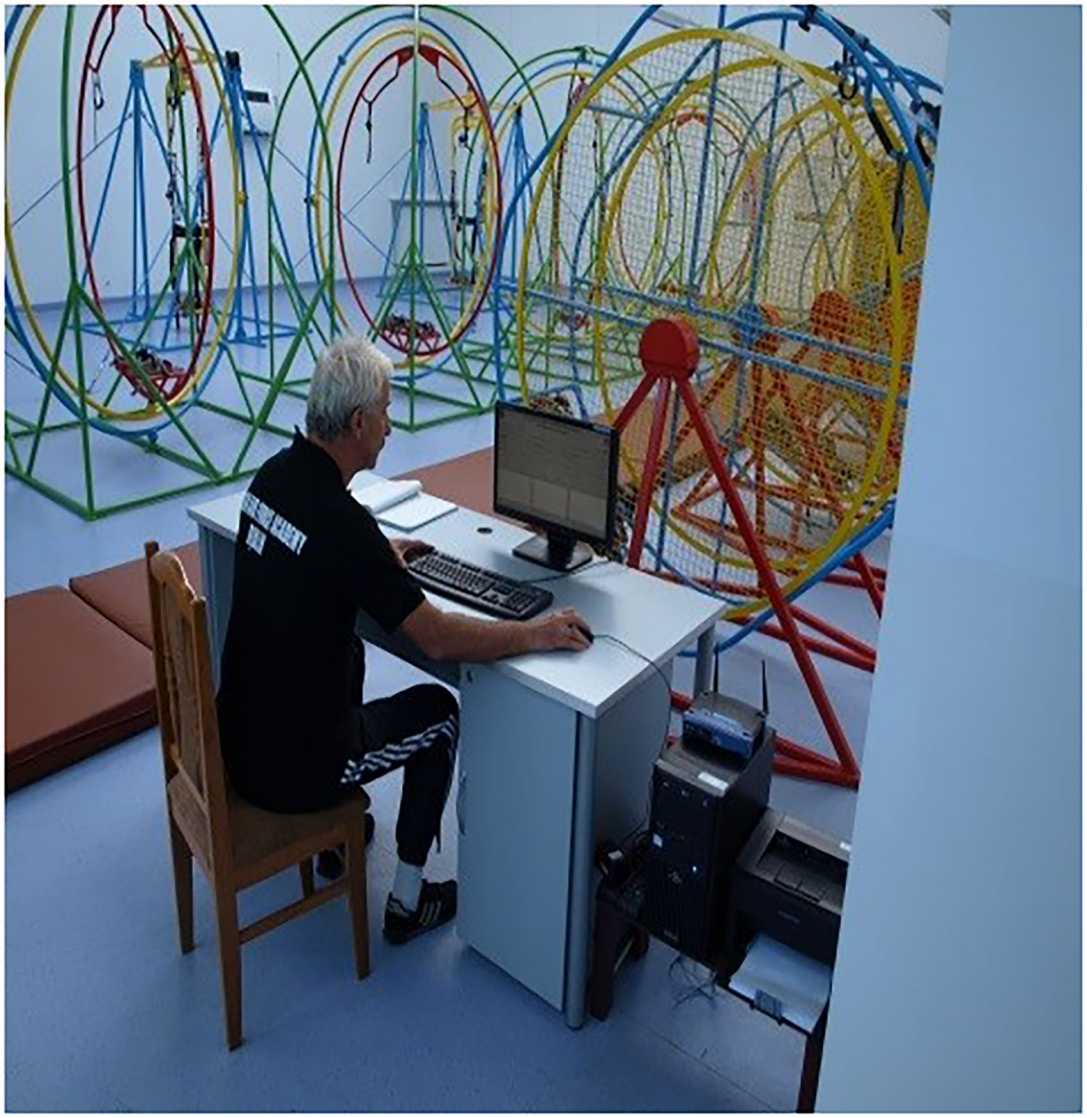
Operator’s station.

The test person was wearing a diagnostic and training device (Figure 1), consisting of a backpack and special glasses (opaque). The test person was attached to a looping swing (arm and leg mount). The test person set the looping swing in motion with his own muscles. The start of the test began when the test person was parallel to the ground. The backpack contained a small computer to communicate the tasks to be performed by the trainee in time standard. The tasks were sent wirelessly from the computer, from the operator (Figure 2) using a relay station, directly to the small computer. The test person was to solve 5 tasks: counting paratroopers in the same colour, counting cars in the same colour, solving arithmetic operations, counting shapes of one type, counting shapes in the same colour. All tasks were displayed in the central field of vision. In addition, immediately after the end of the test, 6th task was performed - a synthetic memory test. Each subsequent test person had a changed order and content of task display. A person at the operator’s station had a preview of the correct answers, marked the correctness of the test person’s answers and controlled the test time (Wochyński et al., 2010a). The tasks and answers were archived by the test operator, at the operator’s station using the diagnostic and training device, immediately after the end of the test. After the end of the test, the operator printed out the report, which showed the test results in percentages and recorded the number of reels made forward. The whole test time was 128 s.

### Training program

The training program on the Special Aviation Gymnastics Instruments included 40 training hours and was divided into 3 parts. The first one covered 26 h and concerned teaching and improving individual exercises. The second part included 8 h of teaching and improving team exercises. The third part was 6 h long, focused on individual spatial orientation and its improvement, with the use of the air environment visualisation system on SAGI. The training program was carried out in the zone of metabolic - aerobic changes. Such a structure of the training process was to ensure adaptation to specific coordination motor skills under the pilot working conditions (Wochyński et al., 2010a). Special pilot training was carried out over 80 days.

### Statistical analysis

Descriptive statistics were used for calculating the arithmetic mean and standard deviation for tests I and II. Results of tests I and II were analyzed for normal distribution using Kolmogorov–Smirnov test, skewness and kurtosis. The r-Pearson correlation between all tested variables was calculated. The difference of results between tests I and II was calculated by analysis of variance (ANOVA) with repeated measurements using the Tukey HSD post-hoc test for pairwise comparison. Effect sizes were calculated using Cohen’s d and interpreted as low (*d* = 0.20 to 0.49), moderate (*d* = 0.50 to 0.79), and high (*d* > 0.80; Cohen, 1988). The obtained values were considered statistically significant when p was less than 0.05. Statistical analysis was performed using the Statistica 13.3 program. The G * Power program was used to assess the sample size (Faul et al., 2007). For evaluate the sample size with the size effect f2 = 0.25 was assume an alpha error of 0.05 and a test’s power of 0.80. The required size of the total sample was estimated at 25 people. Due to the length of the training process, the final analysis was included 20 people.

## Results

Descriptive statistics were used for calculating the arithmetic mean, Kolmogorov–Smirnov test, skewness and kurtosis, Cohen d for tests I and II (Tables 1–4). A statistically insignificant increase in blood pressure and a decrease in heart rate before and after the training process was found (Table 5).

**Table 1:** Elements of normal distribution in somatic and hemodynamic data before and after the training process in cadets pilots (*n* = 20).

Variable					Before training process	After training process			
	M ± SD	K-S	Skewness	Kurtosis	M ± SD	K-S	Skewness	Kurtosis
Age [years]	20.8 ± 1.30	0.31	2.61	6.84	21.1 ± 1.29	0.31	2.61	6.84
Body height [cm]	177.30 ± 7.54	0.11	0.54	1.71	177.3 ± 7.52	0.11	0.52	1.76
Body weight [kg]	72.21 ± 8.09	0.11	0.19	−0.88	73.99 ± 9.1	0.11	0.23	−0.85
BMI [kg/m^2^]	22.95 ± 1.96	0.12	0.18	−0.69	23.5 ± 2.41	0.14	0.48	−0.37
Systolic pressure before test [mm Hg]	135.3 ± 12.37	0.14	0.73	0.61	140.5 ± 10.07	0.12	−0.04	0.79
Diastolic pressure before test [mm Hg]	78.85 ± 10.87	0.10	0.21	0.009	84.3 ± 11.32	0.14	0.94	1.44
Systolic pressure after test [mm Hg]	152.8 ± 18.10	0.14	−0.89	1.25	158.1 ± 18.97	0.13	0.32	−0.31
Diastolic pressure after test [mm Hg]	86.4 ± 18.10	0.09	0.61	0.50	90.05 ± 13.41	0.18	0.79	−0.26
HR before test [bpm]	84.65 ± 14.68	0.14	1.06	1.28	80.7 ± 16.68	0.13	1.01	2.10
HR after test [bpm]	116.4 ± 13.56	0.12	0.17	1.44	112.7 ± 15.38	0.14	0.31	0.34

M ± SD—Mean, Standard Deviation; K-S-Kolmogorov–Smirnov test.

**Table 2:** Difference in somatic data and in hemodynamic parameters during the psychomotor test before and after the training process of cadet pilots (*n* = 20).

Variable	Before training process M ± SD	After training process M ± SD	Cohen’s d test	F	p
Age [years]	20.8 ± 1.30	21.1 ± 1.29	0.23	0.000	1.00
Body height [cm]	177.30 ± 7.54	177.38 ± 7.52	0.01	0.0009	0.98
Body weight [kg]	72.21 ± 8.09	73.99 ± 9.1	0.20	0.42	0.52
BMI [kg/m^2^]	22.95 ± 1.96	23.5 ± 2.41	0.25	0.62	0.43
Systolic pressure before test [mm Hg]	135.35 ± 12.37	140.5 ± 10.07	0.45	2.08	0.16
Diastolic pressure before test [mm Hg]	78.85 ± 10.87	84.3 ± 11.32	0.49	2.41	0.13
Systolic pressure after test [mm Hg]	152.85 ± 18.10	158.1 ± 18.97	0.28	0.80	0.37
Diastolic pressure after test [mm Hg]	86.4 ± 18.10	90.05 ± 13.41	0.23	0.97	0.33
HR before test [bpm]	84.65 ± 14.68	80.7 ± 16.68	0.25	0.63	0.43
HR after test [bpm]	116.4 ± 13.56	112.7 ± 15.38	0.25	0.65	0.42

M ± SD—Mean, standard deviation; p-value of the difference.

**Table 3:** Elements of normal distribution in individual psychomotor skills before and after the training process in cadets pilots (*n* = 20).

Variable					Before training process	After training process			
	M ± SD	K-S	Skewness	Kurtosis	M ± SD	K-S	Skewness	Kurtosis
Percentage ability to complete the task [%]	65.6 ± 24.51	0.21	−0.23	−0.85	86.4 ± 13.05	0.20	−0.37	−1.13
Counting paratroopers in the same colour [%]	80.0 ± 0.41	0.48	−1.62	0.69	90.0 ± 0.31	0.52	−2.88	7.03
Counting cars in the same colour [%]	75.0 ± 0.44	0.46	−1.25	−0.49	95.0 ± 0.22	0.53	−4.47	20.00
Arithmetic actions [%]	40.0 ± 0.50	0.38	0.44	−2.01	75.0 ± 0.44	0.46	−1.25	−0.49
Counting shapes of one type [%]	70.0 ± 0.47	0.43	−0.94	−1.24	90.0 ± 0.31	0.52	−2.88	7.03
Counting shapes in the same colour [%]	85.0 ± 0.37	0.50	−2.12	2.77	95.0 ± 0.22	0.53	−4.47	20.00
Synthetic memory test [%]	50.0 ± 0.51	0.33	0.000	−2.23	75.0 ± 0.44	0.46	−1.25	−0.49
Number of completed reels	32.55 ± 21.97	0.18	−0.30	−1.55	40.2 ± 20.06	0.25	−0.50	−0.60

M ± SD—Mean, standard deviation; K-S-Kolmogorov–Smirnov test.

**Table 4:** Difference in individual psychomotor skills before and after the training process in cadets pilots (*n* = 20).

Variable	Before training process M ± SD	After training process M ± SD	Cohen’s d test	F	p
Percentage ability to complete the task [%]	65.6 ± 24.51	86.4 ± 13.05	1.06	11.22	<0.01
Counting paratroopers in the same colour [%]	80.0 ± 0.41	90.0 ± 0.31	0.27	0.76	0.38
Counting cars in the same colour [%]	75.0 ± 0.44	95.0 ± 0.22	0.57	3.23	0.08
Arithmetic actions [%]	40.0 ± 0.50	75.0 ± 0.44	0.74	5.44	<0.05
Counting shapes of one type [%]	70.0 ± 0.47	90.0 ± 0.31	0.50	2.53	0.12
Counting shapes in the same colour [%]	85.0 ± 0.37	95.0 ± 0.22	0.32	1.08	0.30
Synthetic memory test [%]	50.0 ± 0.51	75.0 ± 0.44	0.52	2.71	0.11
Number of completed reels	32.55 ± 21.97	40.2 ± 20.06	1.35	1.32	0.26

M ± SD—Mean, standard deviation; p-value of the difference.

**Table 5:** Somatic data and haemodynamic parameters during the psychomotor test before and after the training process in cadet pilots.

Variable	Before training Process	After training Process	Significance value
Age [years]	20.8 ± 1.30	21.1 ± 1.29	0.97
Body height [cm]	177.3 ± 7.54	177.38 ± 7.52	0.98
Body weight [kg]	72.21 ± 8.09	73.99 ± 9.1	0.52
BMI [kg/m^2^]	22.95 ± 1.96	23.5 ± 2.41	0.43
Systolic pressure before test [mm Hg]	135.35 ± 12.37	140.5 ± 10.07	0.16
Diastolic pressure before test [mm Hg]	78.85 ± 10.87	84.3 ± 11.32	0.13
Systolic pressure after test [mm Hg]	152.85 ± 18.10	158.1 ± 18.97	0.38
Diastolic pressure after test [mm Hg]	86.4 ± 18.10	90.05 ± 13.41	0.33
HR before test [bpm]	84.65 ± 14.68	80.7 ± 16.68	0.43
HR after test [bpm]	116.4 ± 13.56	112.7 ± 15.38	0.42

During the second test, a statistically significant (at *p* < 0.01) percentage increase in the ability to perform all tasks in relation to the first test was found. Among the specified tasks, a statistically significant increase in the correctness of arithmetical actions was observed in the second test in relation to the first one (with *p* < 0.05). It was shown that the remaining tasks and the number of completed reels in the second test improved, but they were not statistically significant (Table 6).

**Table 6:** Percentage ability to complete all tasks during the forward reels before and after the training process in cadet pilots.

Variable	Before training Process	After training process	Significance value
Percentage ability to complete the task [%]	65.6 ± 24.51	86.4 ± 13.05	*p* < 0.01
Counting paratroopers in the same colour [%]	80 ± 0.41	90 ± 0.31	0.39
Counting cars in the same colour [%]	75 ± 0.44	95 ± 0.22	0.08
Arithmetic actions [%]	40 ± 0.50	75 ± 0.44	*p* < 0.05
Counting shapes of one type [%]	70 ± 0.47	90 ± 0.31	0.12
Counting shapes in the same colour [%]	85 ± 0.37	95 ± 0.22	0.30
Synthetic memory test [%]	50 ± 0.51	75 ± 0.44	0.11
Number of completed reels	32.55 ± 21.97	40.2 ± 20.06	0.26

The results indicate many statistically significant changes in the relationships before and after the training process (Table 7 and Table 8). In the first test (before the training process), it was shown that the number of reels performed correlates negatively and statistically significant with age (at *p* < 0.01) and positively with the percentage ability to perform all tasks during the test (at *p* < 0.05). The percentage ability to perform all tasks in the test positively correlates with three tasks included: counting paratroopers in one colour (with *p* < 0.05), arithmetic actions (with *p* < 0.01), as well as counting shapes of one type (with *p* < 0.01). Moreover, it positively correlates with the number of reels made forward (with *p* < 0.05) and negatively with age (with *p* < 0.05). The task of counting paratroopers in the same colour showed a statistically significant negative correlation with age (with *p* < 0.01) and a positive correlation with the percentage ability to perform all tasks during the test (with *p* < 0.05) and counting shapes of one type (with *p* < 0.05). The arithmetic task showed a significant positive correlation with the diastolic pressure measured before the test (with *p* < 0.01). The task consisting in counting shapes of one type showed a positive correlation with the percentage ability to perform all tasks (with *p* < 0.01) and counting paratroopers of the same color (with *p* < 0.05) and negative correlation with age (with *p* < 0.01). Synthetic memory test showed a positive, statistically significant correlation with systolic pressure after the test (with *p* < 0.01) and heart rate before the test (with *p* < 0.05). The age of the respondents showed a statistically significant negative correlation with the number of completed reels (with *p* < 0.01), the percentage ability to perform all tasks during the test (with *p* < 0.05), as well as the tasks consisting of counting one type of shapes (with *p* < 0.01) and counting shapes of the same colour (with *p* < 0.05).

**Table 7:** Correlations between age. Hemodynamic ratios. Number of reels and individual test tasks before the training process.

Variable	1	2	3	4	5	6	7	8
1	X							
2	*r* = 0.45 *p* < 0.05	X						
3	*r* = 0.37 *p* = 0.11	*r* = 0.50 *p* < 0.05	X					
4	*r* = 0.41 *p* = 0.07	*r* = 0.38 *p* = 0.10	*r* = 0.29 *p* = 0.22	X				
5	*r* = 0.17 *p* = 0.47	*r* = 0.67 *p* < 0.01	*r* = 0.15 *p* = 0.52	*r* = 0.24 *p* = 0.32	X			
6	*r* = 0.35 *p* = 0.13	*r* = 0.74 *p* < 0.01	*r* = 0.49 *p* < 0.05	*r* = −0.13 *p* = 0.60	*r* = 0.31 *p* = 0.18	X		
7	*r* = 0.29 *p* = 0.21	*r* = 0.37 *p* = 0.10	*r* = 0.14 *p* = 0.56	*r* = 0.08 *p* = 0.74	*r* = 0.06 *p* = 0.81	*r* = 0.34 *p* = 0.15	X	
8	*r* = 0.03 *p* = 0.91	*r* = 0.39 *p* = 0.09	*r* = −0.25 *p* = 0.29	*r* = −0.12 *p* = 0.63	*r* = 0.20 *p* = 0.39	*r* = 0.22 *p* = 0.36	*r* = −0.14 *p* = 0.56	X
9	*r* = −0.57 *p* < 0.01	*r* = −0.51 *p* < 0.05	*r* = −0.67 *p* < 0.01	*r* = −0.20 *p* = 0.39	*r* = 0.06 *p* = 0.81	*r* = −0.57 *p* < 0.01	*r* = −0.49 *p* < 0.05	*r* = 0.08 *p* = 0.74
10	*r* = −0.15 *p* = 0.52	*r* = 0.15 *p* = 0.51	*r* = −0.19 *p* = 0.41	*r* = −0.34 *p* = 0.14	*r* = 0.37 *p* = 0.11	*r* = 0.18 *p* = 0.44	*r* = 0.49 *p* < 0.05	*r* = 0.04 *p* = 0.88
11	*r* = −0.15 *p* = 0.54	*r* = 0.33 *p* = 0.15	*r* = 0.12 *p* = 0.61	*r* = 0.01 *p* = 0.96	*r* = 0.62 *p* < 0.01	*r* = 0.12 *p* = 0.60	*r* = 0.14 *p* = 0.56	*r* = −0.03 *p* = 0.89
12	*r* = 0.19 *p* = 0.42	*r* = 0.34 *p* = 0.15	*r* = −0.01 *p* = 0.96	*r* = −0.17 *p* = 0.48	*r* = 0.30 *p* = 0.20	*r* = 0.20 *p* = 0.39	*r* = 0.15 *p* = 0.54	*r* = 0.60 *p* < 0.01
13	*r* = 0.29 *p* = 0.22	*r* = 0.21 *p* = 0.38	*r* = −0.06 *p* = 0.81	*r* = 0.12 *p* = 0.61	*r* = 0.18 *p* = 0.44	*r* = 0.11 *p* = 0.65	*r* = 0.40 *p* = 0.08	*r* = 0.02 *p* = 0.93
14	*r* = −0.23 *p* = 0.32	*r* = 0.36 *p* = 0.12	*r* = −0.07 *p* = 0.76	*r* = −0.22 *p* = 0.34	*r* = 0.33 *p* = 0.16	*r* = 0.30 *p* = 0.19	*r* = 0.13 *p* = 0.59	*r* = 0.45 *p* < 0.05
15	*r* = 0.40 *p* = 0.08	*r* = 0.41 *p* = 0.07	*r* = 0.39 *p* = 0.09	*r* = 0.43 *p* = 0.06	*r* = 0.11 *p* = 0.63	*r* = 0.19 *p* = 0.42	*r* = −0.01 *p* = 0.97	*r* = 0.18 *p* = 0.44

1, Number of completed reels; 2, Percentage ability to complete the task; 3, Counting paratroopers in the same colour; 4, Counting cars in the same colour; 5, Arithmetic actions; 6, Counting shapes of one type; 7, Counting shapes in the same colour; 8, Synthetic memory test; 9, Age; 10, Systolic pressure before test; 11, Diastolic pressure before test; 12, Systolic pressure after test; 13, Diastolic pressure after test; 14, HR before test; 15, HR after test; *r*, Correlation value; *p*, Significance value.

**Table 8:** Correlations between age, hemodynamic ratios, number of reels and individual test tasks after the training process.

Variable	1	2	3	4	5	6	7	8
1	X							
2	*r* = 0.33 *p* = 0.15	X						
3	*r* = 0.23 *p* = 0.34	*r* = 0.31 *p* = 0.18	X					
4	*r* = 0.19 *p* = 0.42	*r* = 0.37 *p* = 0.11	*r* = −0.08 *p* = 0.75	X				
5	*r* = 0.12 *p* = 0.62	*r* = 0.31 *p* = 0.19	*r* = −0.19 *p* = 0.42	*r* = −0.13 *p* = 0.58	X			
6	*r* = 0.15 *p* = 0.53	*r* = 0.53 *p* < 0.05	*r* = −0.11 *p* = 0.64	*r* = −0.08 *p* = 0.75	*r* = 0.19 *p* = 0.42	X		
7	*r* = 0.19 *p* = 0.42	*r* = 0.37 *p* = 0.11	*r* = −0.08 *p* = 0.75	*r* = 1.00 *p* = −--	*r* = −0.13 *p* = 0.58	*r* = −0.08 *p* = 0.75	X	
8	*r* = 0.01 *p* = 0.98	*r* = 0.46 *p* < 0.05	*r* = 0.19 *p* = 0.42	*r* = −0.13 *p* = 0.58	*r* = −0.33 *p* = 0.15	*r* = 0.19 *p* = 0.42	*r* = −0.01 *p* = 0.58	X
9	*r* = −0.50 *p* < 0.05	*r* = −0.19 *p* = 0.42	*r* = −0.35 *p* = 0.13	*r* = 0.13 *p* = 0.58	*r* = 0.19 *p* = 0.41	*r* = −0.11 *p* = 0.65	*r* = 0.13 *p* = 0.58	*r* = −0.34 *p* = 0.14
10	*r* = −0.24 *p* = 0.30	*r* = −0.16 *p* = 0.49	*r* = −0.41 *p* = 0.07	*r* = 0.11 *p* = 0.66	*r* = 0.06 *p* = 0.786	*r* = −0.37 *p* = 0.11	*r* = 0.11 *p* = 0.66	*r* = 0.09 *p* = 0.71
11	*r* = −0.20 *p* = 0.39	*r* = −0.30 *p* = 0.20	*r* = −0.29 *p* = 0.21	*r* = 0.01 *p* = 0.98	*r* = −0.15 *p* = 0.52	*r* = −0.40 *p* = 0.08	*r* = 0.01 *p* = 0.98	*r* = 0.11 *p* = 0.45
12	*r* = −0.05 *p* = 0.82	*r* = 0.26 *p* = 0.27	*r* = −0.06 *p* = 0.78	*r* = 0.44 *p* = 0.06	*r* = −0.02 *p* = 0.95	*r* = 0.22 *p* = 0.36	*r* = 0.44 *p* = 0.06	*r* = −0.08 *p* = 0.72
13	*r* = −0.07 *p* = 0.79	*r* = −0.16 *p* = 0.49	*r* = 0.12 *p* = 0.63	*r* = 0.07 *p* = 0.77	*r* = −0.17 *p* = 0.46	*r* = 0.01 *p* = 0.95	*r* = 0.07 *p* = 0.77	*r* = −0.27 *p* = 0.25
14	*r* = 0.28 *p* = 0.22	*r* = 0.10 *p* = 0.69	*r* = −0.09 *p* = 0.71	*r* = 0.09 *p* = 0.69	*r* = 0.02 *p* = 0.92	*r* = 0.07 *p* = 0.78	*r* = 0.09 *p* = 0.69	*r* = 0.06 *p* = 0.80
15	*r* = 0.17 *p* = 0.48	*r* = 0.17 *p* = 0.49	*r* = 0.26 *p* = 0.27	*r* = −0.23 *p* = 0.32	*r* = −0.13 *p* = 0.59	*r* = 0.13 *p* = 0.59	*r* = −0.23 *p* = 0.32	*r* = 0.38 *p* = 0.10

1, Number of completed reels; 2, Percentage ability to complete the task; 3, Counting paratroopers in the same colour; 4, Counting cars in the same colour; 5, Arithmetic actions; 6, Counting shapes of one type; 7, Counting shapes in the same colour; 8, Synthetic memory test; 9, Age; 10, Systolic pressure before test; 11, Diastolic pressure before test; 12, Systolic pressure after test; 13, Diastolic pressure after test; 14, HR before test; 15, HR after test; *r*, Correlation value; *p*, Significance value.

The results obtained during the second test (after the end of the training process) showed a negative, statistically significant correlation between the number of reels made and age (at *p* < 0.05; Table 8). A statistically significant positive correlation was observed between the percentage ability to complete all tasks and counting one type of shape (with *p* < 0.05) and a synthetic memory test (with *p* < 0.05; Table 8). It was found that the percentage ability to complete all tasks occurs in the same correlation with shape counting as before the training process, but it is at a lower statistical significance level. The other variables showed no statistically significant correlation (Table 8).

## Discussion

On the basis of the results obtained in the sample after the training process (test II), an increase in physical skills was found in the number of completed reels, an increase in correct answers in tasks such as: counting parachutists in the same colour, counting cars in the same colour, counting shapes of one type, counting shapes of the same colour, synthetic memory test and an increase in the values of indicators such as age, systolic and diastolic pressure before and after the test, heart rate before and after the test, compared to the pre-training period (test I), but not statistically significant. However, a statistically significant difference was found in the percentage of ability to perform all tasks and in the task of arithmetic operations. It follows that the percentage ability to complete all tasks is closely related to the number of completed reels. It was observed from the course of the tests that the respondents performing a larger number of reels made more mistakes in answering the questions asked in the central field of vision. With a smaller number of completed reels, they achieved a higher percentage of ability to complete all tasks. The reason for the feedback between the number of reels and the percentage ability to complete all tasks during the test is the specificity of the reels performed on the looping. During these exercises, there are positive +Gz (head - legs direction) and negative -Gz (head - legs direction) accelerations in the tested body, which may contribute to lower efficiency in answering the questions. Similar conclusions were made in tests, carried out using a human centrifuge, concerning the acceleration tolerance level. It has been shown that an increased level of loading causes a delay in response time and a delay in response to visual stimuli (Truszczynski et al., 2014). The results obtained by the subjects depend on the rate of fatigue during exercise and the level of exercise adaptation. Taking into account the results obtained by the respondents in the number of completed reels on the looping and the percentage ability to complete the task [%], it can be concluded that the completed training process increased their level of psychomotor performance, as evidenced by the value of the Cohen’s test effect (Table 4).

The tests showed an increase in the number of completed reels and the percentage of ability to complete all tasks. This shows a higher level of psychomotority, after the training process on SAGI, compared to test I. The improvement of psychomotority is achieved by means of two subsystems - motor and sensory (Lisowski and Mihuta, 2013). As a result of the training process the level of integration of the subsystems increased. Barron and Rose (2017) showed that pilots with a higher level of multitasking achieve higher performance in flight (tested by math tasks, memorizing and monitoring). They recommended multitasking as a predictor for the selection of future military pilots. This is confirmed by a significant correlation showed in authors study between the percentage ability to complete all tasks and the number of completed reels in test I and the lack of statistical significance in test II, which is characterised by higher results in both the number of completed reels and the percentage ability to complete all tasks (psychomotor level). This may be explained by the fact that a decrease in the correlation value (feedback) between the two subsystems in test II is associated with an increase in the level of psychomotority and the level of multitasking of the tested, cadet - pilots.

In these tests, it is interesting to note that age shows significant correlations in both test I and test II. In test I with the number of completed reels, the percentage ability to perform all tasks during the test and the tasks included in the test, such as counting parachutists in the same colour, counting shapes of one type and counting shapes of the same colour. In test II, it shows a correlation only with the number of reels and it is at a lower level of significance, which may indicate an increase in psychomotor skills despite the increase in age. In the previous studies it has been proved many times that the level of psychomotor skills is strongly related to age and the level of difficulty of tasks performed (Armbruester et al., 2007; Sutter and Oehl, 2010; Tan and Sun, 2021). Considering the identical training process and the same conditions of everyday functioning of the test persons, these results indicate a positive effect of the training programme on SAGI on the level of psychomotor skills.

The percentage ability to perform all tasks during test I was significantly correlated with its components, such as counting paratroopers of the same colour, arithmetic activities and counting shapes of one type. In test II, significance was demonstrated with counting of one type of shapes and a synthetic memory test. What is important, attention should be paid to the decrease in the number of statistically significant correlations and their level of significance in test II, as compared to test I. This may indicate an increase in the level of psychomotority, which results in a decrease in the influence of individual components on the percentage ability to perform all tasks. It should be emphasized that the test persons in tests I and test II performed physical effort in the zone of aerobic metabolism, as evidenced by HR values. Also noteworthy is the pre-exercise increase in blood pressure. This may be due to pre-exercise stress and lack of knowledge of the training device (test I) and the desire to improve the previously obtained result (test II). The higher blood pressure found at the end of test II may be due to an increase in the number of reels, which may be associated with a longer effect of this exercise on blood system receptors. Similar indicators were analyzed in studies on performing various tasks on aviation simulators. These studies showed that heart rate and blood pressure levels are influenced by the difficulty level of the pilot’s task. In addition, the difficulty of the tasks and the amount of visual information causes a decrease in the number of movements (blinking eyes) and their duration. It has been demonstrated that mental effort increases arterial pressure and heart rate (Veltman and Gaillard, 1996). Similar conclusions were reached by Leino et al. (1999), analyzing neuroendocrine responses and psychomotority in the selection process of candidates for military pilots. Comparing the test procedure to an air mission, they proved that it is characterized by a high level of mental strain. Moreover, they showed that low neuroendocrine responses in the psychomotor test were associated with good stress tolerance. It is important, however, that both these tests, unlike ours, were not characterized by physical strain. These studies show that raising blood pressure is not only the result of physical effort, but it can also be raised by mental strain.

So far, pilots were tested before and after the training process was completed, however, in resting conditions (without connection with physical exercise; Leino et al., 1999; Russo et al., 2005; Tomczak, 2015; Tomczak et al., 2017). The authors of these studies have demonstrated that their tests have diagnostic value in terms of physical and mental skills under normal and extreme working conditions of a military pilot. Realization an aerial mission in extreme conditions for a long time may lead to disorder motor skills. That observations were made by Tomczak et al. (2019) on the soldiers of the Polish Army. Therefore, the authors believe that for military pilots, the psychological test should be performed under strain conditions.

Based on the results of the tests, it has been shown that the training program on SAGI has a great influence on increasing psychomotor skills in cadet pilots. The diagnostic and training device used during the looping test gave an opportunity to assess attention concentration, reaction and psychophysical condition under extreme environmental conditions. The test combined with a diagnostic and training device was found to be highly useful in the process of special pilot training.

Moreover, the conducted pilot studies will be helpful in assessing future studies that will be carried out before and after aviation practices in the air. As a reference point, it will allow to assess the impact of stress occurring in conditions of real flight in pilots on the level of them psychomotoricity. Comparing the speed of response to the questions (stimulus) and the speed of execution of reels on forward on looping in a limited time can provide valuable guidance for practical use in pilot training.

## Conclusion

The authors found that a special training process on SAGI had a positive effect on the level of psychomotority in cadet pilots, by increasing the percentage of the task capacity measured by the diagnostic and training device and the number of reels performed in the test after the training process (test II) compared to the test before the training process (test I). After the training process (test II), a reduction in the positive correlation between the ability to perform tasks [%] and the number of forward turns on looping compared to test I was found. According to the size of the Cohen’ d effect the training process influenced at psychomotor skills and percentage ability to complete the task at high level improved.

## Data availability statement

The raw data supporting the conclusions of this article will be made available by the authors, without undue reservation.

## Ethics statement

The studies involving human participants were reviewed and approved by the researchers have obtained the consent of the Bioethics Committee, at the Medical University of Poznań, issued on 15 May 2019 with the number 610/19. The patients/participants provided their written informed consent to participate in this study.

## Author contributions

AP and ZW contributed to conception and design of the study. AP organized the database. AP and ZW performed the statistical analysis. AP wrote the first draft of the manuscript. AP wrote sections of the manuscript. All authors contributed to the article and approved the submitted version.

## Conflict of interest

The authors declare that the research was conducted in the absence of any commercial or financial relationships that could be construed as a potential conflict of interest.

## Publisher’s note

All claims expressed in this article are solely those of the authors and do not necessarily represent those of their affiliated organizations, or those of the publisher, the editors and the reviewers. Any product that may be evaluated in this article, or claim that may be made by its manufacturer, is not guaranteed or endorsed by the publisher.
